# Dual foraging and pair coordination during chick provisioning by Manx shearwaters: empirical evidence supported by a simple model

**DOI:** 10.1242/jeb.120626

**Published:** 2015-07-01

**Authors:** Akiko Shoji, Stéphane Aris-Brosou, Annette Fayet, Oliver Padget, Christopher Perrins, Tim Guilford

**Affiliations:** 1 Oxford University, Department of Zoology, South Parks Road, Oxford OX1 3PS, UK; 2 University of Ottawa, Department of Mathematics, Ottawa, Ontario, CanadaK1N 6N5

**Keywords:** Foraging strategy, *Puffinus puffinus*, Bio-logging, GPS, Diving

## Abstract

The optimal allocation of time and energy between one's own survival and offspring survival is critical for iteroparous animals, but creates a conflict between what maximises the parent's fitness and what maximises fitness of the offspring. For central-place foragers, provisioning strategies may reflect this allocation, while the distance between central-places and foraging areas may influence the decision. Nevertheless, few studies have explored the link between life history and foraging in the context of resource allocation. Studying foraging behaviour alongside food load rates to chicks provides a useful system for understanding the foraging decisions made during parent–offspring conflict. Using simultaneously deployed GPS and time–depth recorders, we examined the provisioning strategies in free-living Manx shearwaters *Puffinus puffinus*, which were caring for young. Our results showed a bimodal pattern, where birds alternate short and long trips. Short trips were associated with higher feeding frequency and larger meals than long trips, suggesting that long trips were performed for self-feeding. Furthermore, most foraging was carried out within 100 km of sea fronts. A simple model based on patch quality and travel time shows that for Manx shearwaters combining chick feeding and self-maintenance, bimodal foraging trip durations optimise feeding rates.

## Abstract (precis)

**Summary:** A simple model based on patch quality and travel time shows that for Manx shearwaters combining chick feeding and self-maintenance, bimodal foraging trip durations optimise feeding rates.

## INTRODUCTION

Resource allocation between parents and their offspring during reproduction is a central issue in life-history theory (Ydenberg et al., 1994; McNamara and Houston, 1997) and the outcomes of parent–offspring conflict are inextricably linked with fitness (Nur, 1988). In iteroparous species, life-history theory predicts that individuals should balance the cost of their own survival and future reproductive success against investment in current reproduction (Stearns, 1992). For instance, current parental effort may be increased in parents in good condition, but decreased in parents in poor condition so that they can maintain their own body condition.

Seabirds are on the extreme slow end of the life-history continuum: marine resources are generally patchily and scarcely distributed, and are assumed to be unpredictable (but see Weimerskirch, 2007), making it difficult for pelagic seabirds to regulate foraging patterns and in particular chick provisioning. Possible mechanisms that control provisioning behaviour in adult Procellariiforms have been reported, but this issue is still contentious and seems to be species specific with no clear phylogenetic pattern. Indeed, while provisioning behaviour is shaped by chick condition in most Procellariiformes such as northern fulmars *Fulmar glacialis* (Hamer and Thompson, 1997), Manx shearwaters *Puffinus puffinus* (Hamer et al., 1999), yellow-nosed albatrosses *Thalassarche chlororhynchos* (Weimerskirch et al., 2000) or wedge-tailed shearwaters *Puffinus pacificus* (Baduini, 2002), it is determined by adult body mass in two species of the *Puffinus* genus: sooty shearwaters *Puffinus griseus* and short-tailed shearwaters *Puffinus tenuirostris* (Weimerskirch, 1998; Weimerskirch and Cherel, 1998). But evidence shows that tight regulation of pair coordination in foraging schedule can be critical in species with bi-parental care to ensure that energy demands of the offspring are met without over-feeding (Harris and Wanless, 2011). However, the relative importance of pair coordination in chick provisioning is still unclear.

Some studies have suggested that feeding rates in pelagic seabirds are fixed by inherent internal rhythms so that parents feed their offspring regardless of offspring condition (Ricklefs, 1992; Hamer and Hill, 1993). Other studies report that parents show more flexibility in feeding rates than previously thought, so that they do modify feeding patterns according to the offspring's condition (Hamer and Hill, 1993; Bolton, 1995; Weimerskirch, 1995; Tveraa et al., 1998). Furthermore, sex-specific differences in chick-provisioning behaviour during the breeding season among monomorphic birds have been found in a number of species (e.g. Hamer et al., 2006; Elliott et al., 2010).

A dual foraging strategy, where parents alternate or mix short and long trips, is one example of how parental seabirds can regulate foraging patterns. During short trips, parents forage at local oceanic shelf areas to maximise offspring provisioning rates because the costs of commuting with a food load for the chick are kept low (Cuthill and Kacelnik, 1990). In contrast, during long trips, parents can travel further to exploit inherently richer but more distant areas of deep oceanic water or seasonally stable sea fronts – boundaries between waters of different density that enhance primary productivity by inducing an upward supply of nutrients (Mahadevan and Archer, 2000). At such hot spots, parents can replenish their own reserves without paying the costs of repeated commuting (Matsumoto, 2008). A disadvantage of executing the long trips for offspring is that feeding rates to offspring are lower because commuting time is longer (Chaurand and Weimerskirch, 1994; Weimerskirch et al., 1994; Weimerskirch, 1998). Furthermore, energetic or nutritional requirements often differ between parents and their young (Murphy, 1996), and thus foraging locations may vary depending on the intended recipient of food (Markman et al., 2004). A number of *Proceraliiform* seabirds execute a bimodal foraging pattern; these include thin-billed prions *Pachyptilla belcheri* (Weimerskirch et al., 1994), yellow-nosed albatrosses *Diomedea chlororynchos* (Pinaud et al., 2005), wandering albatrosses *D. exulans* (Weimerskirch et al., 1994), sooty shearwaters *P. griseus* (Weimerskirch, 1998), little shearwaters *P. assimilis* (Booth et al., 2000), Cory's shearwaters *Calonectris diomedea* (Granadeiro et al., 1998; Magalhães et al., 2008), streaked shearwaters *C. leucomelas* (Ochi et al., 2010), Buller's albatrosses *Thalassarche bulleri* (Stahl and Sagar, 2006) and blue petrels *Halobaena caerulea* (Chaurand and Weimerskirch, 1994), as do a number of *Alcids* [little auks *Alle alle* (Welcker et al., 2009; Brown et al., 2012; Jakubas et al., 2012)] and *Sphenisciformes* [little penguins *Eudyptula minor* (Saraux et al., 2011); Adélie penguins *Pygoscelis adeliae* (Ropert-Coudert et al., 2004)]. Yet dual-foraging strategies are not ubiquitous among seabirds (Phillips et al., 2009). Furthermore, factors affecting the parents' decision to undertake a long or short foraging trip may be species specific. Previous studies have mainly focused on the frequency distribution of trip duration or the use of different foraging habitats, but few studies have connected those variables to meal mass, chick-feeding rates or foraging behaviour at sea (Phillips et al., 2009).

**List of symbols and abbreviations**

EHFC_day_: daily energy gains for chicks
IPQ: index of patch quality
TDR: time–depth recorder
TPQ_25_: time required to accumulate 25 points on the IPQ scale
*t*_T_: travel time

Manx shearwaters (*Puffinus puffinus*) are widely distributed in the North Atlantic Ocean (Brooke, 1990). This species shows a typical Proceraliiforme life-history pattern with a single-egg clutch and slow chick development that averages 70 days until fledging (Brooke, 1990). The species exhibits bi-parental care during both incubation and the chick-rearing period, and colony arrival and departure only occur at night (Riou and Hamer, 2008). Variation in foraging trip durations and foraging destinations during chick-rearing have been reported (Guilford et al., 2008), making shearwaters good candidates for testing foraging strategies from the standpoint of resource partitioning. The purpose of this study was to combine at-sea data (movement patterns and diving behaviour) with at-colony data (breeding schedule, meal mass delivered to young) collected from breeding shearwaters to test for and analyse dual foraging strategies in the context of regulation of provisioning (i.e. fasting duration of offspring, chick growth rates). We develop a simple model describing the energetics of foraging and show that model predictions support our interpretation of the empirical evidence on the importance of dual foraging in chick provisioning.

## RESULTS

We retrieved and successfully downloaded GPS and time–depth recorder (TDR) data from 17 Manx shearwaters (*Puffinus puffinus* Brünnich 1764) birds out of the 22 originally deployed – the other 5 birds returned without a GPS logger. While bio-logging methods may impact behaviour as reported in Phillips et al. (2003), breeding success in our study plot (0.69) was similar to that of the undisturbed plot (0.60) at Skomer Island.

**Fig. 1.**
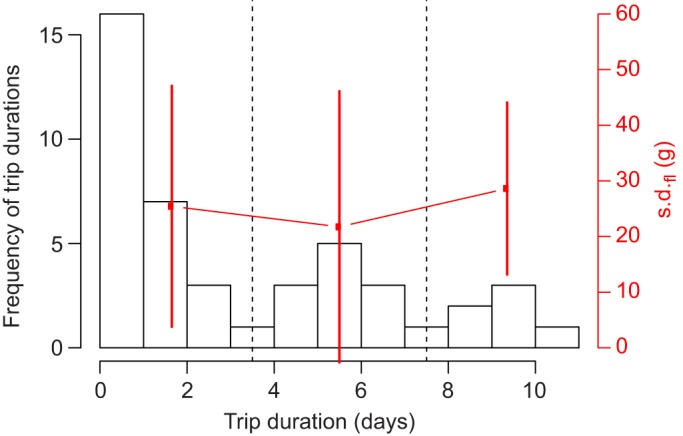
**Density of trip duration during chick-rearing period in the Manx shearwater (*Puffinus puffinus*).** The histogram represents the distribution of trip durations, split into three categories (separated by black vertical broken lines); within each category, the s.d. of food load (in grams) is shown as red dot (right vertical scale); red vertical bars represent 1 s.d., estimated by bootstrapping binned food loads 1000 times.

**Fig. 2.**
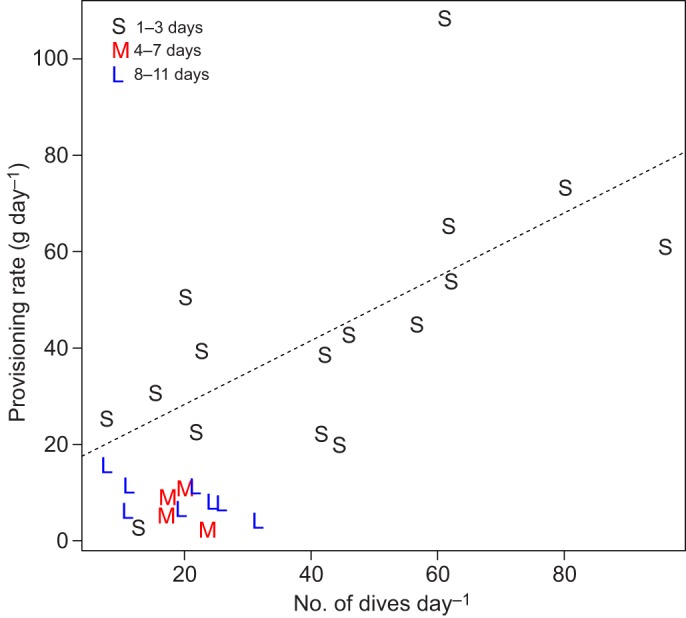
**Distribution of provisioning rate and frequency of dives in chick-rearing Manx shearwaters.** Each symbol indicates one trip type (S: short; M: medium; L: long). The dashed line represents the linear mixed model fitted for short trip durations (S).

GPS recorders logged 15 complete foraging trips from females and 29 complete foraging trips from males during chick rearing. No sex difference in foraging parameters was detected (trip duration: *F*_1,43_=0.729, *P*=0.398; travelled distance: *F*_1,16841_=1.773, *P*=0.183; flight speed: *F*_1,16841_=2.614, *P*=0.106; food load: *F*_1,42_=0.750, *P*=0.391; average trip similarity: *t*=−1.695, d.f.=13.739, *P*=0.113), which is consistent with Dean (2012), and therefore data from both sexes were pooled. Trip duration and total distance travelled per trip were highly correlated (*r*=0.84). The distribution of foraging trip durations showed three peaks, with short trips lasting 1–3 days, medium trips lasting 4–7 days and long trips, 8–11 days (Fig. 1). Mean adult body mass was 417±38 g before and 406±30 g after each trip. Adult body mass was independent of trip durations (ΔAIC=0.6) with body mass before short trips (416±35 g), medium trips (406±42 g) or long trips (437±42 g), or after short trips (407±28 g), medium trips (397±30 g) or long trips (412±44 g). Meal size was similar across the three trip duration types (short trip: 40±4.89 g; medium trip: 50±5.67 g; long trip: 53±12.73 g). Among these three modes of trip duration, however, shorter trips appeared to be significantly more productive, as shown in Fig. 2 because the provisioning rate (meal mass per day) was much higher for 1- or 2-day trips than for longer trips (ΔAIC=−9.27; Fig. 2).

Alternatively, prey quality may trade off with variability in food load, so that extending travel time and distance may increase the chance of finding high quality prey items, which are often rare and therefore less reliably found. This possibility is known as the quality–variability trade-off hypothesis (Litzow et al., 2004). To test this idea, we discretised the distribution of trip durations again into short, medium and long durations and computed the standard deviation of food load (s.d._fl_) within each category. The empirical distribution of s.d._fl_ was estimated by bootstrapping food load observations 1000 times within each category. While s.d._fl_ in the short category was smaller than in the long category, s.d._fl_ in the medium category was the lowest, and error bars across all three bins largely overlapped (Fig. 1), thereby suggesting that the quality–variability trade-off hypothesis does not explain the results.

**Fig. 3.**
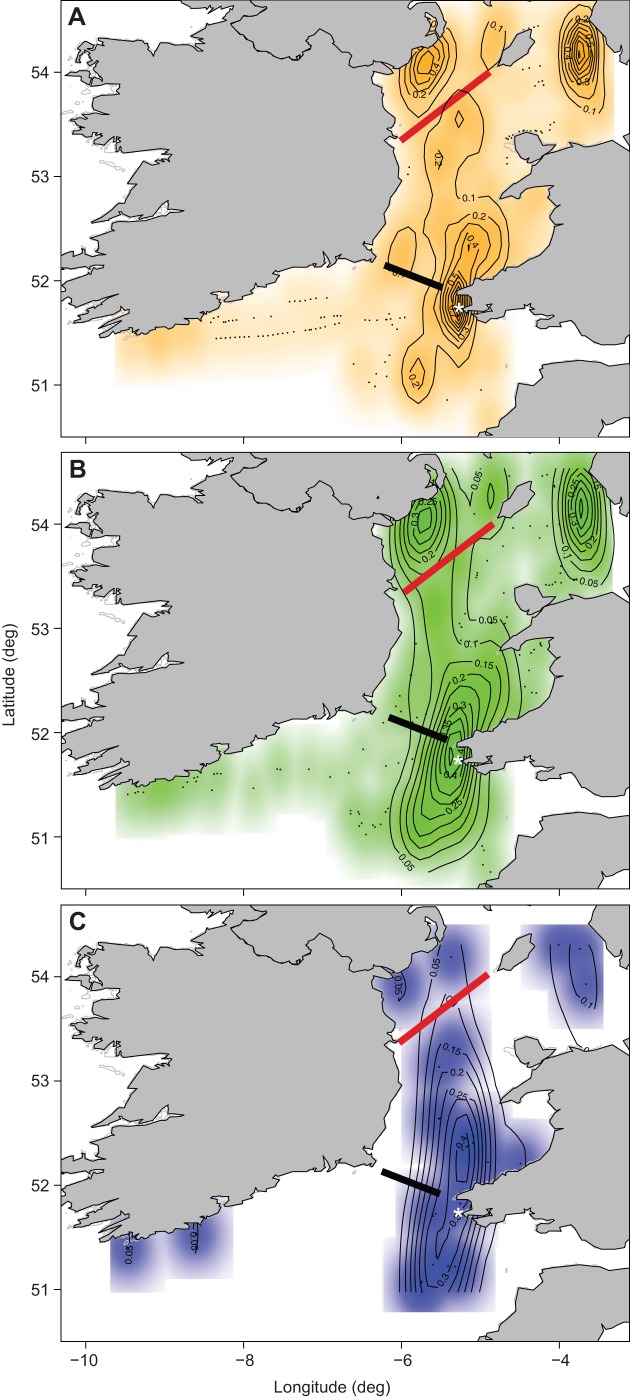
**Contour maps of activities of chick-rearing Manx shearwaters in 2013.** (A) Resting (orange), (B) flying (green) and (C) foraging (blue). The intensity of shading indicates the density of the raw data. The asterisk indicates the position of Skomer Island. The approximate locations of the Irish Sea front (red line) and Celtic Sea front (black line) are shown on each map (after Simpson and Hunter, 1974).

**Fig. 4.**
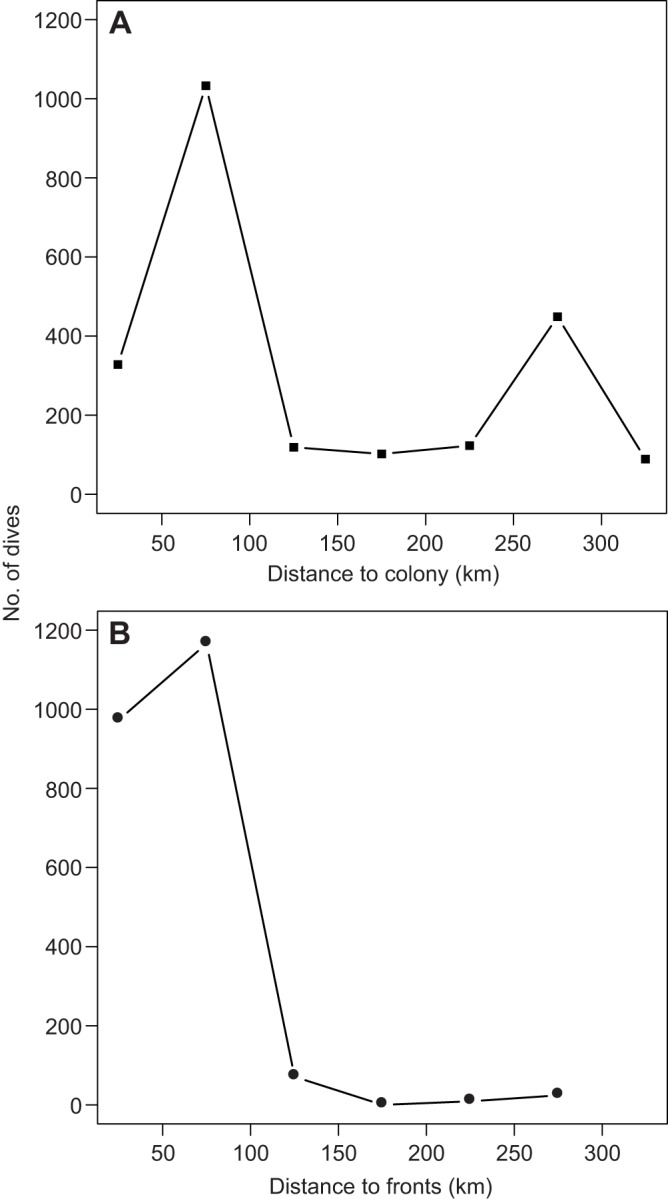
**Dive intensity in relation to colony and fronts.** Number of dives as a function of distance to (A) colony and (B) Irish or Celtic Sea front. In each panel, distances were split in bins of 50 km starting from a distance of 25 km. Number of dives were tallied within each bin.

**Fig. 5.**
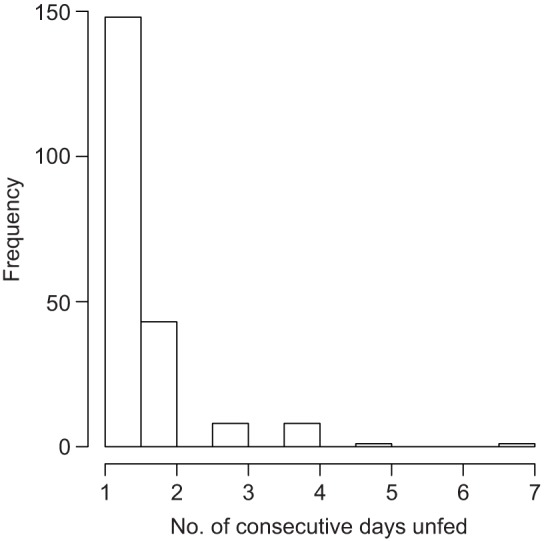
**Frequency of the intervals between feeding of the chick.**

Fig. 3 shows the contour maps of activity patterns in resting, flying and foraging individuals. While both resting and flying behaviours were made at a wide range of locations (Fig. 3A,B), foraging was more highly concentrated around the colony (Fig. 3C). Distance between dive locations (=foraging locations) and the colony showed a clear bimodal pattern (Fig. 4A). In contrast, distance between dive locations and front lines (as shown in Fig. 3) showed a unimodal pattern (Fig. 4B). The number of dives per trip increased slightly but significantly with trip duration (ΔAIC=−13.37), whereas daily number of dives decreased with trip duration; shorter trips had higher number of dives per day (ΔAIC=−5.70). The duration of foraging trips did not affect meal size per trip (ΔAIC=1.68), but provisioning rate (g day^−1^) decreased with trip duration. Daily chick growth rate (from hatching to the last meal) was 6.47±5.22 g and the provisioning period was 63.67±2.77 days (*N*=15). The frequency of the interval between subsequent feeds decreased after 3 days regardless of the starting condition, but at least half of these intervals are less than 2 days (Fig. 5).

### Modelling of dual foraging

**Fig. 6.**
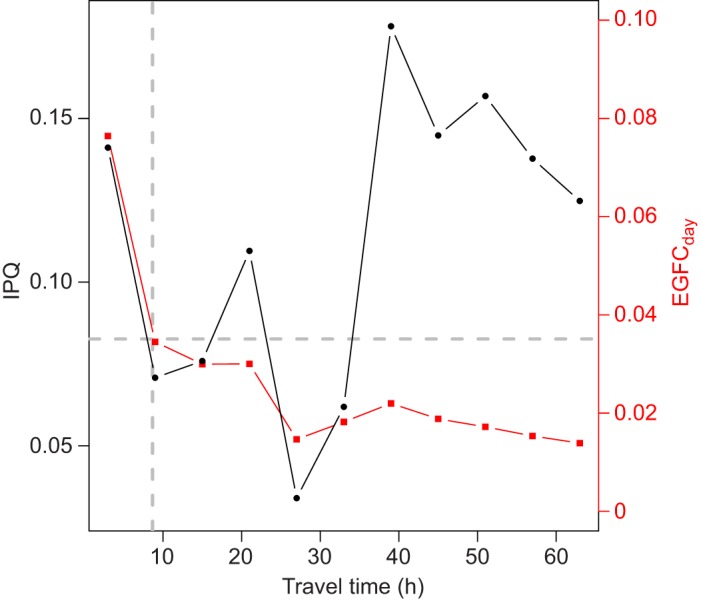
**A model of dual foraging based on index of patch quality in Manx shearwaters.** The black line indicates observed values of IPQ and the red line indicates the estimated daily energy gain for chicks (EGFC_day_) as a function of travel time (h). We assumed that birds had a constant flight speed and that (1) patch quality, measured on the IPQ scale, is gained during 6 hours of foraging; (2) adults only bring back food when they have collected 25 points on the IPQ scale; the time required to accumulate these 25 points is denoted TPQ_25_. EGFC_day_ is inversely proportional to TPQ_25_. The horizontal grey line indicates the values of EGFC_day_ that were reduced to 50% of the maximum value. The vertical grey line indicates where the EGFC_day_ crossed 50% of the maximum value of EGFC_day_.

In our data, Manx shearwaters showed a tri-modal distribution of trip durations under visual inspection (Fig. 1). To relate this foraging pattern to provisioning, we estimated daily energy gains for chicks (EGFC_day_), which we plotted as a function of travel time in parallel with estimated index of patch quality (IPQ). Our modelling results (Fig. 6) show that IPQ as a function of travel time is indeed tri-modal, as in Fig. 1, and that EGFC_day_ is 50% of its maximum value only for short trips (<10 h; Fig. 6). Importantly, this result is robust to the choice of constants used in our modelling (supplementary material Fig. S2).

## DISCUSSION

Central-place foraging theory predicts that animals should use distant foraging patches only when energy intake rate increases with distance from the colony (Charnov, 1976; Ropert-Coudert et al., 2004). While variation in duration of foraging trips is well known in pelagic seabirds, few studies have investigated its relationship with meal size to offspring in wild animals (Wanless et al., 1993; Ainley et al., 1998). Here, we show that Manx shearwaters performed a dual foraging strategy in the sense that some trips (the short ones) are for chick provisioning whereas longer trips are for self (Fig. 2). Shearwaters did not increase the meal size delivered to the chick with the travel time. Instead, young shearwaters gained more energy per time unit as adults brought more food back to the chick when they performed short trips. In contrast to other Procellariiformes, which feed young on a partly digested diet that can contain liquid oil (up to 50%), Manx shearwaters deliver little digested food during both short and long trips (Brooke, 1990). As the average number of dives and meal mass delivered to chicks per day decreased with increasing trip length, chicks did not benefit directly from longer trips. We also showed that the quality–variability trade-off hypothesis (Litzow et al., 2004) does not explain the dual foraging pattern.

Foraging mode change-over was not initiated by parents reaching the critical lower body mass, but rather appeared to be coordinated, because chicks were constantly fed by parents (the interval between feeding was mostly 1 or 2 days) and chicks were rarely left unfed for more than 3 days (Fig. 5). We speculate that such a dual-foraging strategy is a consequence of a partner's long trip duration, suggesting that pair-coordination during chick-provisioning shapes foraging patterns, as is the case during incubation change-overs (Brooke, 1990).

Some pelagic seabirds are known to employ a dual-foraging strategy where parents alternate frequent short trips and a single long trip to meet the energetic demands of offspring while maintaining their own condition (Granadeiro et al., 1998; Welcker et al., 2009). For example, Cory's shearwaters use flexible foraging trip durations and parents increase body mass after long trips (Granadeiro et al., 1998). Here, shearwaters changed foraging areas between short and long trips, but in both cases, the foraging areas were highly restricted to an area close to a sea front (Fig. 4). Thus, birds adjusted both trip duration and foraging locations in relation to the demands of offspring or themselves. This raises a question as to why birds use two foraging patches instead of foraging only at the nearby patch, given that travel distance to those patches greatly differ. The area of the Irish Sea where shearwater parents performed long trips is known to be a ‘hot spot’ for seabirds (Begg and Reid, 1997), and lies to the north and west of the Irish Sea front (Pollock et al., 1997; Dean et al., 2012). As a front, this region is expected to be highly productive (Mahadevan and Archer, 2000) and possibly more so than the Celtic Sea. Indeed, IPQ is highest at the distant foraging area in our study. Thus, it is likely that birds increased travel distance to forage at this better foraging site during long trips. One potential explanation for the dual strategy we see here, then, is that short trips lead to foraging near the colony, in areas highly exploited that lead to steady but average-to-low rewards, whilst longer trips are taken once chick provisioning is done to forage in farther off areas, where fishing may become more unpredictable with distance but is potentially of higher reward (Weimerskirch, 2007).

In support of this explanation, our model demonstrates that net rate of gain per day decreases with distance and travel time for chicks, but increases for foraging parents themselves, suggesting an advantage of foraging nearby to the colony for chicks. However, we did not find that parents increased their body mass after long trips. Rather, the body mass was similar after both short and long trips. We do not have a definitive answer as to why parents did not increase their body mass if they performed long trips to maintain their own body condition. A possible explanation for the contradiction is that during long trips shearwaters foraged on better quality food (e.g. more oil), which would not have been immediately detectable using mass measurements alone (Einoder et al., 2013). Future studies could benefit from evaluating the energetic content of prey throughout the chick-rearing period to examine whether variation in foraging strategies is associated with variation in prey quality. Moreover, increasing body mass would also increase the cost of travelling (Kacelnik and Cuthill, 1990). Manx shearwaters use intermittent flapping flight for the long-range trips (Spivey et al., 2014) and thus, not only meal size, but also adult body mass, may be modulated to minimise travel cost because flight costs must increase with body mass.

## MATERIALS AND METHODS

### Study site and birds

The study was carried out at Skomer Island (51°44′N, 5°17′W), Wales, UK, during a single breeding season (July–August 2013) to avoid potentially confounding effects of inter-annual environmental variability. All study birds were ringed as part of the long-term monitoring program carried out by Oxford University since 2006. Parents were sexed where possible by cloacal inspection during the laying period (Gray and Hamer, 2001). Nests were visited daily to monitor breeding progress (laying dates, hatching dates, fledging dates where possible). All chicks at the monitored burrows in the colony were weighed daily. To determine food load from parents to their chicks, we weighed chicks every evening at 8pm before adult shearwaters arrived at the colony and checked study burrows every 20 min through the night (typically between 23:00 h–04:00 h). To reduce disturbance, we used knock-down sticks at the entrance (Shoji and Gaston, 2010), only checking nests when sticks were displaced. When we found an adult in a study burrow, we blocked the nest and left at least 20 min to allow parents to feed young before weighing both parent and chick. All work was conducted after ethical approval by the Countryside Council for Wales, the Skomer Island Advisory Committee and the British Trust for Ornithology (BTO permits: T.G., 5311; C.P., 660; A.S., 5939).

### Foraging behaviour

To study the foraging behaviour of chick-rearing shearwaters, we simultaneously deployed 1 Hz CEFAS G5 TDRs (sampling interval=1 s, recording duration=7 or 14 days) weighing 2.7 g, attached to a hand-made darvic leg ring, and GPS loggers (sampling interval=5 min, recording duration range=1–11 days; unpackaged i-gotU GT-120: Mobile Action, New Taiwan City, Taiwan) weighing 10–12 g, fitted dorsally to each bird using Tesa tape underlying a small group of contour feathers (Guilford et al., 2008) on 14 males and 8 females from the study colony for 1–7 successive foraging trips. Birds were taken from study burrows by hand through a short access tunnel and weighed at device deployment and retrieval. Handling time for capture and retrieval was always less than 15 min.

### Data analysis

All analyses were performed in R (R Development Core Team, 2014). We quantified trip duration, total distance travelled and foraging range (the maximum distance from the colony). All positional fixes were converted to metres using the Universal Transverse Mercator coordinate system. Horizontal ground speed was calculated from interpolated positions by using cubic splines of GPS position fixes.

To monitor diving behaviour, we used diveMove (Luque and Fried, 2011), which corrected for device drift. We obtained dive depth, duration and surface pause duration for all dives and determined bouts based on sequential differences (Mori et al., 2001). Only dives deeper than 1 m were analysed because shallow dives are often associated with non-foraging behaviour, such as bathing or socialising.

Activity was determined by combining GPS and TDR data: GPS-recorded speeds were used to determine ‘flying’ when birds were moving faster than 5 km h^−1^ (supplementary material Fig. S1; see also Guilford et al., 2008); TDR-detected dives as per diveMove indicated ‘foraging’; the remainder of the time (speed<5 km h^−1^; no dives) was classified ‘resting’. Positions of seasonally stable fronts (Celtic Sea Front and Western Irish Sea Front) were obtained from the literature (Simpson and Hunter, 1974) to examine effects of the foraging locations in shearwaters as shown in Scales et al. (2014).

Analysis of average trip similarity was based on the nearest neighbour analysis (NNA; Freeman et al., 2011). For this, trip information was extracted from the GPS data, a foraging trip starting when the bird flies outside of a 2 km radius around the colony and ending when it comes back within this radius. Because we were not interested in homing behaviour but in foraging behaviour, only outbound trips were used. These are defined by the period between the start of each trip and the point along the route that is most distant from the colony. Route similarity between two trips is then computed by the match point distance, which is the sum of the minimum distances between each positional fix along a focal trip versus a comparison trip. The resulting distance matrix is symmetrised by taking the average match point distance between each pair of trips. The average trip similarity for each trip is computed by taking the row (or column) average. These averages for males and females were then compared with Welch's *t*-test.

We used an information theoretic approach to evaluate the relationship between (1) trip duration and (2) locations, and provision rates (g day^−1^), and number of dives per day, meal mass per trip and total number of dives per trip. All analyses were completed using linear mixed models with a maximum-likelihood fitting method allowing for inter-model comparisons with the lme4 package R (Bates and Maechler, 2009). Data were collected more than once from individuals and so to account for pseudo-replication, individual identity was included as a random effect in the models (Buckley et al., 2003). Model selection was based on Akaike's information criterion (AIC) and ΔAIC from the null model (intercept-only). Means are presented as ±1 standard deviation unless otherwise stated. We checked for deviations from normality and homoscedasticity by plotting fitted and observed values and residuals.

### Dual-foraging modelling

We aimed to identify dual foraging in the Manx shearwater by describing how food load size varies as a function of travel time by modifying the model presented in Ropert-Coudert et al. (2004). When the travel time increases (from short to long), it is expected that the food load maximising provisioning rate should also increase (Charnov, 1976) to balance energy gain against expenditure. When seabirds exhibit a dual-foraging mode, it is likely that provisioning occurs during short trips, while self-feeding takes place during long trips: indeed, the cost of loading food is expected to increase with flight time and distance (Kacelnik, 1984). Here, we used the index of patch quality (IPQ) as a proxy for prey richness estimated based on the dive profiles (Mori et al., 2002; Shoji et al., 2014). The rationale behind IPQ is as follows. During foraging, it is expected that parameters associated with dive profiles (e.g. duration of diving, descending, ascending as well as bottom and surface times) reflect prey richness (Mori et al., 2002). This assumes that patch residence time in diving animals should be positively correlated with both travel time from surface to a patch and patch richness, in order to maximise energy intake per units of time (Stephens and Krebs, 1986). Although the accuracy of the index is relatively rough because of the noise inherent in data logger, IPQ has been shown to reflet prey richness in diving animals (thick-billed murres *Uria lomvia*: Mori et al., 2002; Elliott et al., 2008, Weddel seals *Leptonychotes weddellii*: Mori et al., 2005). Detailed descriptions for the calculation of IPQ are available in Mori et al. (2002) and Elliott et al. (2008).

Taking inspiration from modelling done by Ropert-Coudert et al. (2004), we examined how variation in travel time [which is highly correlated (*r*=0.84) to travel distance] affects the rate of energy gain by adults and chicks. We assumed that birds had a constant flight speed (derived empirically: see supplementary material Fig. S1) and that (1) patch quality, measured on the IPQ scale, is gained during 6 h of foraging; (2) adults only bring back food when they have collected 25 points on the IPQ scale; the time required to accumulate these 25 points is henceforth denoted TPQ_25_. Daily energy gain for chicks (EGFC_day_) is inversely proportional to the time to gain (TPQ_25_):
(1)
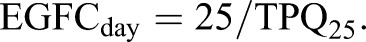

In turn, the time to gain 25 IPQ points includes travel time *t*_T_ plus foraging time. Foraging time is inversely proportional to IPQ, as it is expected that prey are easy to forage in a high-quality patch, so that:
(2)
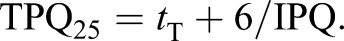

Altogether, we expect that EGFC_day_ is inversely proportional to *t*_T_. From the empirical estimation of IPQ, we can then relate foraging strategy to both travel time and provisioning. As the choice of the constants (6 h; 25 points) is arbitrary, we examined the robustness of our predictions to these values.

## Supplementary Material

Supplementary Material
