# Supplementary material for: Dual foraging and pair coordination during chick provisioning by Manx shearwaters: empirical evidence supported by a simple model
Source: J Exp Biol. 2015 Jul 1;218(13):2116–23. doi: 10.1242/jeb.120626 (PMC4510841; doi:10.1242/jeb.120626)
Supplement: Supplementary Material [file supp_218_13_2116__index.html]

Supplementary Material 

# Dual foraging and pair-coordination during chick provisioning by Manx shearwaters: empirical evidence supported by a simple model

## JEB120626 Supplementary Material

- Supplementary Material
