# Supplementary material for: Dual foraging and pair coordination during chick provisioning by Manx shearwaters: empirical evidence supported by a simple model
Source: J Exp Biol. 2015 Jul 1;218(13):2116–23. doi: 10.1242/jeb.120626 (PMC4510841; doi:10.1242/jeb.120626)
Supplement: Supplementary Material [file supp_jeb.120626_JEB120626supp.pdf]

Supplementary figures

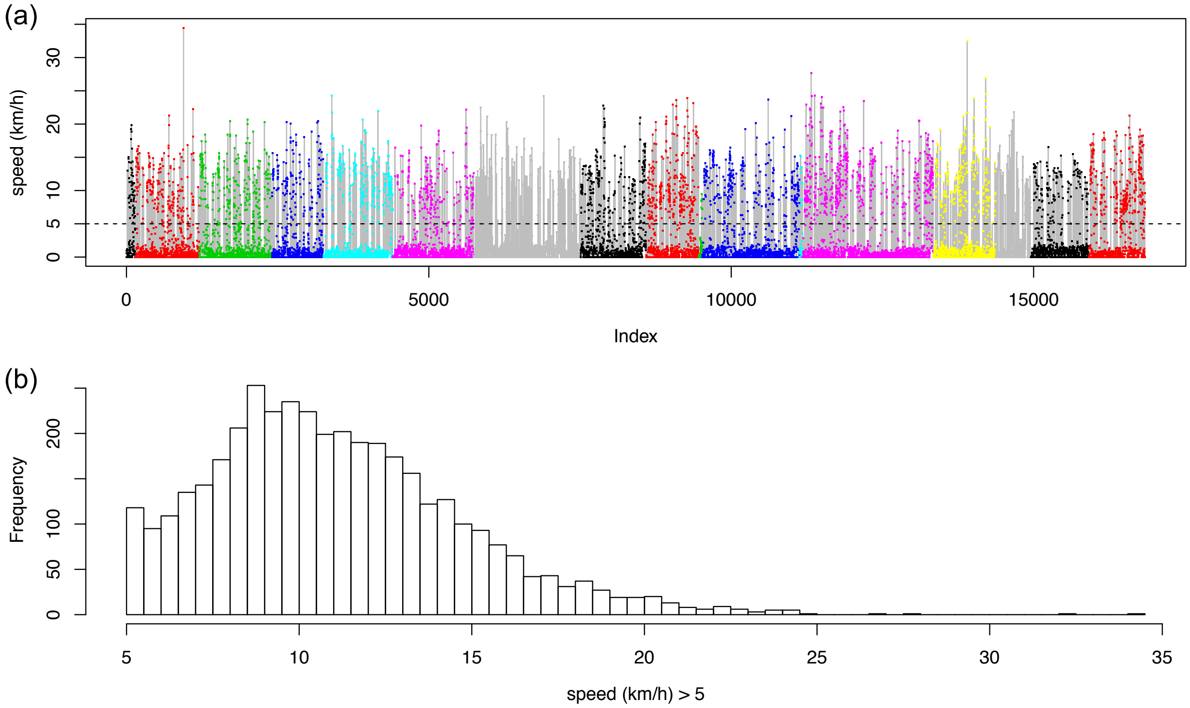

Figure S1: Manx shearwater movement speeds. (a) Trace of speeds recorded by the GPS loggers. Individual birds are identified by alternating colours. (b) Distribution of horizontal surface speed during foraging trips of razorbills ($N = 17$ birds, $n = 44$ trips; only speeds exceeding 5 km/h are considered – see broken horizontal line in panel (a). The black broken line in (a) indicates the cut-off value of 5 km/h used as a flight threshold in this study.

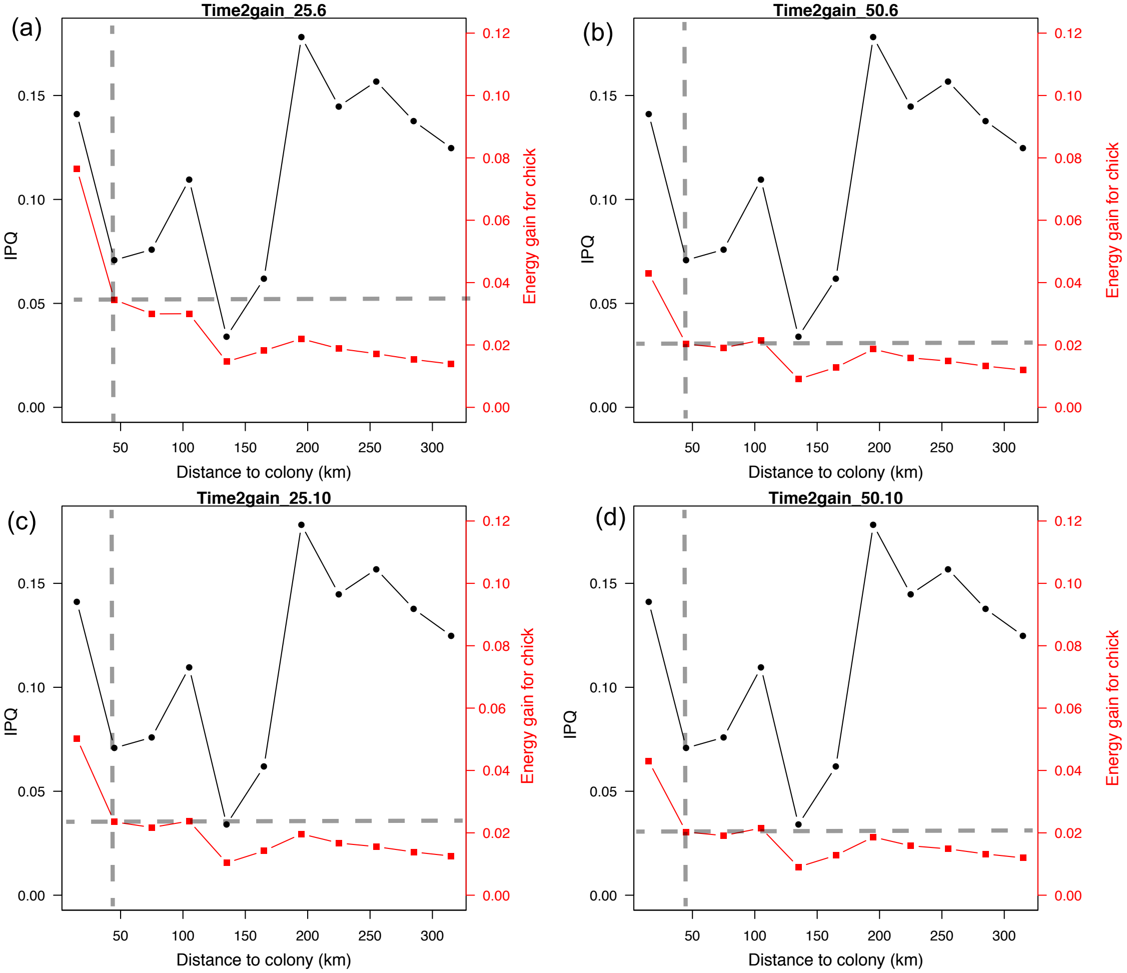

Figure S2: Robustness to assumptions of our IPQ-based model for explaining dual foraging in Manx shearwaters. Black lines indicate observed values of IPQ and red line indicates estimated daily energy gain for chicks ($EGFC_{day}$), both as a function of distance to colony (or equivalently, travel time). In the main text, we assumed that birds had a constant flight speed and that: (i) patch quality, measured on the IPQ scale, is equivalent to six hours of foraging; (ii) adults only bring back food when they have collected 25 points on the IPQ scale; the time required to accumulate these 25 points is henceforth denoted TPQ_{25}. We here show that our results are robust to our choice for the time to gain of 25 points as well as 50 points. Similarly, we presented that patch quality, measured on the IPQ scale, is equivalent to six hours of foraging as well as ten hours of foraging: (a) TPQ_{25} with six hours of foraging; (b) TPQ_{50} with six hours of foraging; (c) TPQ_{25} with ten hours of foraging; (d) TPQ_{50} with ten hours of foraging. Horizontal grey broken lines indicate the values of $EGFC_{day}$ that were reduced to 50% of the maximum values. Vertical grey broken lines indicate the first IPQ mode. In all four cases (panels), the first IPQ mode delivers at least 50% of daily energy gain to chicks.
